# Supplementary material for: Evaluation of the Efficiency in Public Health Centers in Greece Regarding the Human Resources Occupied: A Bootstrap Data Envelopment Analysis Application
Source: Int J Environ Res Public Health. 2022 Jan 30;19(3):1597. doi: 10.3390/ijerph19031597 (PMC8834954; doi:10.3390/ijerph19031597)
Supplement: Supplementary file 1 [file ijerph-19-01597-s001.zip › ijerph-1554780-supplementary.pdf]

Table S1: Mean efficiency scores of each of the 155 health centers for all models

a/a	*M0*		*M1*		*M2*		*M3*		*M4*	
DMU's	*CRS*	*VRS*	*CRS*	*VRS*	*CRS*	*VRS*	*CRS*	*VRS*	*CRS*	*VRS*
1	1	1	0.724	1	0.384	1	0.403	1	0.95	1
2	1	1	0.466	0.511	0.418	0.515	0.642	0.727	0.829	1
3	1	1	0.434	0.464	0.387	0.429	0.639	0.689	0.805	0.855
4	1	1	0.25	0.271	0.305	0.352	0.579	0.631	0.691	1
5	0.848	0.873	0.484	0.573	0.53	0.694	0.5	0.56	0.581	0.683
6	1	1	0.673	1	0.722	1	0.637	1	0.706	1
7	1	1	1	1	1	1	1	1	1	1
8	1	1	0.505	1	0.437	1	0.91	1	1	1
9	1	1	0.845	1	0.536	1	0.827	1	0.44	1
10	0.852	0.865	0.304	0.304	0.669	0.693	0.493	0.506	0.24	0.292
11	1	1	0.739	0.756	0.462	0.48	0.524	1	0.248	0.26
12	0.653	1	0.119	0.333	0.288	0.445	0.653	0.877	0.072	0.098
13	1	1	0.695	1	1	1	0.798	1	1	1
14	1	1	0.738	1	0.824	1	0.763	1	1	1
15	0.899	1	0.423	0.531	0.694	0.716	0.518	0.536	0.199	0.215
16	1	1	0.532	0.544	0.998	1	0.647	0.65	0.241	0.34
17	0.301	0.74	0.137	0.5	0.189	0.407	0.12	0.171	0.153	0.347
18	0.483	0.955	0.195	0.5	0.445	0.671	0.193	0.206	0.225	0.307
19	0.806	1	0.57	1	0.58	0.657	0.559	0.56	0.112	0.128

20	0.733	0.745	0.404	0.41	0.671	0.673	0.514	0.526	0.246	0.288
21	1	1	0.921	1	1	1	0.953	1	0.386	1
22	0.301	1	0.292	1	0.182	0.269	0.175	0.209	0.113	0.141
23	1	1	0.386	0.478	0.805	1	0.995	1	0.517	0.711
24	0.867	0.961	0.385	0.405	0.784	0.784	0.639	0.646	0.185	0.193
25	1	1	0.378	0.396	0.457	0.57	1	1	0.465	0.52
26	0.904	1	0.463	0.747	0.526	0.784	0.658	1	0.284	0.492
27	0.418	0.628	0.301	0.5	0.248	0.32	0.308	0.319	0.081	0.107
28	1	1	1	1	0.438	0.932	0.804	1	0.45	0.711
29	0.484	0.549	0.332	0.363	0.39	0.406	0.27	0.287	0.23	0.234
30	1	1	1	1	0.939	1	1	1	0.655	1
31	0.461	0.783	0.236	0.5	0.419	0.495	0.21	0.212	0.157	0.228
32	0.548	0.752	0.45	0.605	0.4	0.721	0.302	0.366	0.231	0.37
33	0.409	0.539	0.195	0.333	0.338	0.384	0.208	0.22	0.158	0.175
34	1	1	0.443	0.5	1	1	0.281	0.282	0.536	0.57
35	0.846	0.861	0.326	0.355	0.728	0.745	0.173	0.19	0.231	0.267
36	1	1	1	1	0.807	1	0.49	0.586	0.406	0.458
37	0.799	0.8	0.501	0.521	0.694	0.753	0.567	0.579	0.183	0.231
38	1	1	0.598	0.622	0.229	0.236	0.355	0.415	1	1
39	1	1	1	1	0.904	1	1	1	0.948	1
40	1	1	1	1	0.743	0.893	0.99	1	0.563	0.754
41	0.819	0.887	0.758	0.883	0.267	0.276	0.358	0.511	0.357	0.395

42	1	1	0.485	0.525	0.916	1	0.456	0.473	0.622	0.646
43	0.243	0.26	0.153	0.154	0.149	0.149	0.171	0.18	0.087	0.093
44	0.942	1	0.135	0.25	0.279	0.334	0.261	0.267	0.854	1
45	1	1	0.427	0.527	0.8	0.819	0.539	0.543	0.426	0.44
46	0.763	1	0.635	1	0.563	0.572	0.332	0.344	0.267	0.281
47	0.993	1	0.451	0.511	0.866	0.899	0.239	0.244	0.611	0.614
48	0.931	0.985	0.446	0.531	0.899	0.9	0.157	0.163	0.215	0.241
49	1	1	1	1	0.713	0.808	0.579	0.749	0.535	0.556
50	0.995	0.997	0.446	0.527	0.329	0.338	0.688	0.722	0.898	0.922
51	1	1	0.568	0.569	1	1	0.263	0.285	0.366	0.43
52	1	1	1	1	0.505	1	0.552	1	0.621	1
53	0.358	0.551	0.238	0.5	0.235	0.282	0.286	0.295	0.155	0.2
54	1	1	0.278	0.5	0.481	0.589	0.268	0.287	0.835	0.939
55	1	1	1	1	1	1	0.499	1	0.395	1
56	0.889	0.894	0.552	0.574	0.429	0.45	0.651	0.741	0.198	0.239
57	1	1	0.817	1	1	1	0.941	1	0.474	1
58	1	1	0.661	1	1	1	1	1	0.939	1
59	1	1	0.771	0.913	0.543	0.78	0.592	0.666	0.852	1
60	1	1	0.569	0.623	1	1	0.508	0.824	0.297	0.303
61	1	1	0.923	1	1	1	0.567	1	0.556	1
62	0.939	1	0.771	1	0.412	0.413	0.334	0.337	0.338	0.357
63	1	1	1	1	0.32	0.346	0.322	0.36	0.799	0.802

64	0.75	1	0.588	1	0.606	0.643	0.222	0.233	0.274	0.275
65	1	1	0.868	0.893	1	1	0.605	0.625	0.562	0.572
66	0.844	1	0.703	1	0.527	0.593	0.199	0.203	0.19	0.19
67	0.354	1	0.333	1	0.179	0.286	0.087	0.093	0.112	0.25
68	0.73	0.805	0.556	0.564	0.273	0.274	0.485	0.511	0.41	0.411
69	0.731	0.802	0.308	0.334	0.615	0.646	0.211	0.213	0.477	0.487
70	0.557	0.617	0.274	0.333	0.222	0.235	0.115	0.115	0.393	0.422
71	0.586	0.708	0.287	0.5	0.193	0.229	0.538	0.541	0.191	0.221
72	1	1	1	1	1	1	0.234	0.244	0.225	0.225
73	0.815	0.949	0.672	0.794	0.309	0.521	0.386	0.427	0.37	0.632
74	1	1	1	1	1	1	0.804	0.966	0.314	0.482
75	0.893	0.956	0.154	0.2	0.579	0.579	0.439	0.441	0.265	0.298
76	0.845	0.877	0.427	0.508	0.423	0.449	0.394	0.396	0.491	0.53
77	0.61	1	0.5	1	0.36	0.413	0.351	0.364	0.363	0.364
78	1	1	0.11	0.112	0.513	0.524	0.616	0.7	0.717	0.843
79	1	1	0.196	0.262	1	1	0.272	0.621	1	1
80	0.381	1	0.312	1	0.359	0.57	0.225	0.252	0.077	0.137
81	0.559	1	0.174	1	0.197	0.5	0.3	0.5	0.388	1
82	0.482	0.558	0.206	0.333	0.432	0.457	0.133	0.137	0.147	0.149
83	0.947	0.986	0.388	0.501	0.947	0.977	0.153	0.154	0.146	0.151
84	0.595	0.666	0.294	0.344	0.571	0.58	0.245	0.267	0.182	0.182
85	1	1	0.274	0.291	0.506	0.506	1	1	0.27	0.272

86	0.887	1	0.5	1	0.361	0.491	0.726	0.829	0.085	0.133
87	0.982	1	0.197	0.213	0.456	0.475	0.343	0.362	0.786	0.871
88	0.526	0.564	0.258	0.333	0.214	0.239	0.225	0.234	0.395	0.396
89	1	1	0.238	0.255	1	1	0.474	0.546	0.62	0.714
90	0.261	1	0.179	1	0.203	0.5	0.108	0.2	0.117	0.333
91	0.296	1	0.21	1	0.236	0.558	0.146	0.234	0.117	0.333
92	0.888	0.957	0.461	0.5	0.842	0.843	0.626	0.714	0.297	0.322
93	0.773	0.953	0.232	0.333	0.542	0.601	0.385	0.388	0.259	0.305
94	1	1	0.567	0.567	0.77	0.777	0.586	0.966	0.644	0.713
95	0.249	0.604	0.065	0.333	0.214	0.456	0.039	0.06	0.073	0.173
96	1	1	0.342	0.5	0.671	0.678	0.402	0.442	0.725	0.768
97	1	1	1	1	0.849	1	0.941	1	0.447	0.495
98	0.753	1	0.103	0.333	0.515	0.821	0.295	0.345	0.274	0.284
99	0.724	1	0.592	1	0.548	0.568	0.246	0.257	0.295	0.323
100	0.947	1	0.922	1	0.539	0.547	0.491	0.579	0.246	0.284
101	1	1	0.32	0.336	0.749	0.768	0.503	0.507	0.441	0.477
102	0.353	1	0.232	1	0.349	0.746	0.131	0.192	0.041	0.1
103	0.346	1	0.237	1	0.266	0.667	0.135	0.182	0.104	0.27
104	1	1	0.33	0.385	0.84	1	0.634	0.868	0.491	0.918
105	0.522	1	0.152	1	0.166	0.5	0.416	1	0.144	0.507
106	1	1	1	1	0.838	1	0.465	1	0.949	1
107	0.715	0.893	0.353	0.5	0.713	0.793	0.214	0.224	0.241	0.273

108	0.939	1	0.471	1	0.861	0.95	0.206	0.208	0.277	0.282
109	0.398	1	0.321	1	0.259	0.423	0.29	0.316	0.076	0.167
110	1	1	0.515	1	0.299	0.331	1	1	0.244	0.309
111	0.988	1	0.623	1	0.778	0.888	0.398	0.463	0.722	0.825
112	1	1	0.509	0.606	1	1	0.351	0.378	0.194	0.215
113	1	1	0.709	0.877	0.863	0.865	0.343	0.356	0.737	1
114	1	1	1	1	0.793	0.802	0.164	0.189	0.398	0.405
115	0.425	0.74	0.143	0.5	0.287	0.432	0.291	0.368	0.144	0.249
116	0.54	1	0.263	1	0.448	0.778	0.467	0.5	0.098	0.182
117	0.642	1	0.61	1	0.517	0.552	0.311	0.322	0.212	0.213
118	1	1	0.629	1	1	1	0.539	1	0.469	1
119	0.805	0.886	0.157	0.167	0.593	0.616	0.337	0.366	0.45	0.47
120	0.422	0.607	0.289	0.5	0.275	0.31	0.206	0.207	0.105	0.13
121	1	1	0.644	0.689	0.694	0.832	0.99	1	0.767	0.813
122	1	1	0.387	0.5	0.711	0.745	1	1	0.219	0.246
123	0.533	0.639	0.289	0.5	0.33	0.387	0.272	0.273	0.211	0.214
124	0.37	0.959	0.113	0.5	0.276	0.518	0.205	0.288	0.274	0.538
125	0.611	0.747	0.252	0.333	0.472	0.496	0.377	0.386	0.297	0.301
126	0.49	0.759	0.252	0.5	0.301	0.378	0.293	0.314	0.19	0.275
127	1	1	1	1	0.618	1	0.406	1	1	1
128	0.724	1	0.564	1	0.537	0.59	0.329	0.336	0.266	0.275
129	1	1	0.316	0.445	0.853	1	0.369	0.458	0.706	0.729

130	0.937	0.984	0.312	0.333	0.482	0.537	0.554	0.554	0.366	0.382
131	1	1	0.435	1	0.312	0.403	1	1	0.228	0.318
132	1	1	0.318	0.426	0.936	1	0.483	0.497	0.996	1
133	1	1	0.226	0.5	0.542	0.78	0.678	0.828	0.156	0.2
134	0.675	0.76	0.282	0.341	0.531	0.538	0.279	0.282	0.165	0.183
135	0.564	0.787	0.223	0.333	0.476	0.508	0.279	0.284	0.221	0.248
136	1	1	0.111	0.2	0.615	0.679	0.566	0.654	0.818	0.855
137	1	1	1	1	0.965	0.967	0.802	1	0.565	0.616
138	0.784	1	0.407	1	0.478	0.578	0.438	0.531	0.226	0.25
139	1	1	1	1	0.85	0.88	0.502	0.56	1	1
140	0.387	0.626	0.103	0.25	0.338	0.471	0.076	0.08	0.166	0.235
141	1	1	0.75	1	0.321	0.353	0.458	0.482	0.753	0.759
142	0.621	1	0.442	1	0.621	0.858	0.186	0.186	0.105	0.167
143	0.498	0.789	0.193	0.5	0.333	0.466	0.209	0.298	0.188	0.255
144	1	1	1	1	1	1	1	1	0.312	1
145	0.859	1	0.456	1	0.363	0.484	0.757	0.78	0.101	0.109
146	1	1	0.455	0.466	0.764	0.771	1	1	0.58	0.744
147	1	1	0.652	0.927	0.536	1	0.95	1	0.29	0.601
148	0.574	0.638	0.335	0.5	0.293	0.324	0.376	0.382	0.276	0.298
149	1	1	0.901	1	0.409	0.448	0.748	0.875	0.442	0.447
150	1	1	0.525	0.537	0.839	0.935	1	1	0.442	0.461
151	1	1	0.515	0.677	1	1	0.821	0.945	0.386	0.869

152	1	1	0.622	0.635	0.39	0.401	0.699	0.776	0.419	0.504
153	1	1	0.593	1	0.47	1	1	1	1	1
154	1	1	0.717	1	0.521	0.55	0.826	0.883	0.408	0.414
155	1	1	1	1	0.681	0.681	0.72	0.775	1	1
**Mean**	**0.82**	**0.932**	**0.499**	**0.69**	**0.579**	**0.681**	**0.491**	**0.571**	**0.421**	**0.518**
